# The theoretical frameworks behind integrated environmental, ecosystem, and economic accounting systems and their classifications

**DOI:** 10.1016/j.eiar.2019.106317

**Published:** 2020-01

**Authors:** Alessandra La Notte, Charles Rhodes

**Affiliations:** a Joint Research Centre (JRC), European Commission, Via E. Fermi 2749, Ispra I-21027, VA, Italy; b TBD Economics, Gaithersburg, MD, USA

**Keywords:** Ecosystem services, Classification, Natural capital, System of National Accounts

## Abstract

The integration of ecosystem services and accounting systems can help different stakeholders understand the economic implications of environmental impacts. Any such integration requires clear understanding of how ecosystem services may match and integrate with traditional accounts. The Experimental Ecosystem Accounts (EEA) of the System of Integrated Environmental and Economic Accounts (SEEA) is developing quickly with applications at different administrative levels. One emerging feature is lack of agreement on conceptual notions and definitions that could reconcile different approaches. Some basic issues can be developed and solved only once a theoretical basis has been established. Since the first step of any application is to identify which ecosystem services to account for, this paper explores whether and to what extent the theoretical frameworks behind ecosystem services classification systems match the theoretical framework behind the SEEA EEA. This attempt first tackles the conceptual framework on the accounting side, then the conceptual framework on the ecosystem services classification side. Combining the two sides, it is possible to visualize matches or mismatches and to infer a few consequences and implications. Ecosystem services classification systems can guide separation of intra-ecosystem processes from final ecosystem services, and help disentangle ecosystem services from benefits, key requirements for integrating accounts.

## Highlights

• The concept of ecosystem services (ES) is a viable tool for impact assessments.
• Integrating ES into policies could be addressed via integrated accounting systems.
• The conceptual frameworks on the accounting and ES classification sides are explored.
• A consistent integration requires disentangling ES from benefits.
• Identifying the ES that users value requires discriminating among different uses.

## 1 Introduction

The role of ecosystem services in Strategic Environmental Assessment (Geneletti, 2011), Environmental and Social Impact Assessment (Rosa and Sánchez, 2015), and Policy Impact Assessment (Helming et al., 2013) has been acknowledged by several sources.

Beyond the environmental impacts, policy-makers need to understand the economic implications of changes to ecosystem service flows and how they affect different stakeholders, such as economic sectors and households. Systematic accounting of the services and incorporation of the benefits could enable decision-makers to measure stakeholders' reliance on ecosystem services and assess the status of the services on a regular basis (Kumar et al., 2013). Following this path requires a clear understanding of how ecosystem services match and integrate with official accounting systems.

In the traditional national economic accounts, based on the System of National Accounts (SNA), no consideration was given either to environmental damage or to ecosystem assets and services. In the early 1990s the United Nations Statistics Division proposed a System for Integrated Environmental and Economic Accounting (SEEA) (Bartelmus et al., 1991) to fill the information gap in the SNA core accounts with a series of satellite accounts to record environmental data in a consistent way. While at the beginning the 1993 SEEA handbook (UN, 1993) focused on the adjustment of existing macro-indicators, the following SEEA 2003 framework comprised several environmental accounting modules (UNSD et al., 2003). The most recent SEEA Central Framework (SEEA CF) is being implemented as an international statistical standard (UN et al., 2014). Next to the SEEA CF, other developments are in progress: the Experimental Ecosystem Accounts (SEEA EEA) and the SEEA Extensions and policy applications. The SEEA EEA (UN et al., 2014; UN, 2017) in particular receives a growing amount of attention and connects to a remarkable number of related initiatives.

At the international level, the World Bank Group leads the Wealth Accounting and the Valuation of Ecosystem Services (WAVES)^1^ partnership, which aims to mainstream natural resources into development planning and national economic accounts.

In Europe the 7th Environment Action Program (EU, 2013) and the EU Biodiversity Strategy (EC, 2011) include objectives to develop natural capital accounting (NCA) in the EU; specifically the Knowledge Innovation Project for Integrated Natural Capital Accounting (KIP INCA)^2^ tackles ecosystem and ecosystem services accounts. Some guidance documents have been made available on the compilation of supply and use tables for ecosystem services accounting (Vallecillo et al., 2018 and La Notte et al., 2017a) and ecosystem extent accounts. KIP-INCA also links member-state activities with the Mapping and Assessment of Ecosystems and their Services (MAES) initiative.^3^

At a national level, the project Advancing the SEEA EEA is promoting tests and applications in seven pilot countries (South Africa, Mauritius, Chile, Mexico, Indonesia, Vietnam, and Buthan). In line with the SEEA, other countries started developing and implementing ecosystem accounting systems, such as Australia with its Environmental Systems Modelling platform (EnSym)^4^; Canada with the project Measuring Ecosystem Goods and Services (MEGS)^5^; and Italy^6^ and the UK^7^ with the establishment and endorsement of the Natural Capital Committee. There are sub-national applications as well: in Australia experimental ecosystem accounts were applied to the central highlands of Victoria (Keith et al., 2016); in the Netherlands, physical and monetary ecosystem accounts were tested for Limburg Province (Remme et al., 2015). Local case studies of accounts for multiple ecosystem services can be found for Norway (Schröter et al., 2014) and Italy (Busch et al., 2012).

One feature that clearly emerges is the lack of common ground on conceptual notions and definitions that reconcile all approaches – major issues include whether it is necessary to separate biophysical things created by ecological processes from ‘final’ ecosystem services (ES),^8^ as discussed in Hein et al. (2015) and implemented in UN (2017); or to separate the definition of ecosystem services from the definition of benefits, where in Mononen et al. (2016) we find an example that uses benefits as ecosystem services. Moreover, since the first step of any application is to identify which ecosystem services to assess, before measurement and valuation, an agreed standard classification for ecosystem services could be very useful. There are a few ecosystem services classification systems that the SEEA EEA experts are currently taking into consideration. Obst (2016) has remarked that relevant measurement concepts first need to be established, and then the components to analyze these concepts can be finalized in a series of classifications. Relevant measurement concepts depend on the underpinning theoretical framework. The question to explore is whether and to what extent the theoretical frameworks behind ecosystem services classification systems match the theoretical framework behind the SEEA EEA. By answering this question, it is possible to start clarifying conceptual notions that currently remain ambiguous, and to start providing some more consistent definitions.

This paper describes first the conceptual framework on the accounting side (Section 2), and then the conceptual frameworks on the ecosystem services classification side (Section 3). Based on the former, there seem to be gaps in clarity in the definitions of SNA and non-SNA benefits (SEEA EEA), and in environmental assets (SEEA CF): a fundamental motivation of this paper is to highlight this issue and to offer a draft possible solution. After attempting to compare features of the accounting and ecosystem services classification frameworks in combination (Section 4), a discussion follows about the consequences of matching or failing to match objectives (Section 5). Section 6 offers some insights for future developments.

## 2 The underpinning theoretical frameworks on the accounting side

### 2.1 Economic accounting framework

**Fig. 1:**
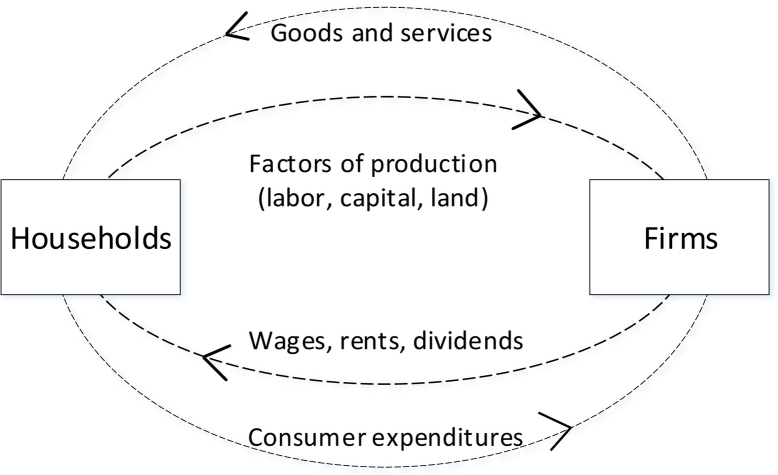
Simplified representation of the circular flow model.

The System of National Accounts (SNA) is grounded in macroeconomics. The conceptual framework that graphically simplifies the way different actors play in the system is the circular-flow model (Fig. 1).

Fig. 1 represents the flows of goods and money within (i) the market for goods and services, where households buy goods and services from firms; and (ii) the market for factors of production, where firms buy factors of production from households.

In (i) households spend their money buying what is produced and sold by firms. In (ii) households offer their labor, capital, and other factors such as land, receiving an income. Firms will in turn use these factors to produce goods and services.

Fig. 1 reports the simplest possible representation: more elaborate representations include factor and product markets, government, financial institutions, imports and exports, and so on. For our purpose, we can stick to the simplest representation and consider that there is something missing from the economic sphere:

• some ‘natural’ inputs (other than labor, capital, and land) that actually enter the production and consumption sphere and should be managed sustainably (e.g., aquatic and water resources, timber, mineral resources);
• some undesired outputs from both production and consumption that enter the environment as residuals (i.e., air emissions, wastewater, solid wastes).

In economics the over-exploitation of natural resources and the emissions of pollutants are typically included within the family of negative ‘externalities’, and represent the cost or diseconomy occurring during the production or the consumption of goods and services that is imposed on non-transacting third parties. Negative externalities do not find a place within the SNA.

### 2.2 SEEA Central Framework

Since its first drafts, the stated purpose of the SEEA is to ‘complete’ the SNA core accounts by adding a series of satellite accounts which should report missing information about natural inputs and residuals, following basic principles and rules in order to provide fully comparable and integrated outcomes consistent with the SNA structure. Satellite accounts could be internal when they detail accounting items that are already in the SNA (e.g., Environmental Protection Expenditures, Environmental Taxes, and more general environmental transactions within the economy), and external when they add accounts that are not in the SNA (e.g., non-produced natural resources, pollutants generated by economic activities).

Within a previous version of the SEEA (ref. paragraph 1.23 in UNSD et al., 2003) natural capital is defined as including three basic categories:

• resource functions which cover natural resources extracted and converted into goods and services (e.g., timber from natural forests, subsoil deposits);
• sink functions which absorb pollution and wastes generated by production and consumption activities (e.g., air emissions, wastewater, solid waste);
• service functions which guarantee habitat for all living beings (e.g., air, water, amenity functions).

Since 2003, the SEEA has evolved remarkably, and those ‘functions’ previously introduced in a shortened way have been developed as an additional framework (i.e., Experimental Ecosystem Accounts, ref. following section). Now the SEEA Central Framework (CF) is being proposed and implemented as a standard framework for integrated environmental accounts, covering measurement in: (i) flows of materials and energy in physical terms; (ii) stocks of environmental assets; and (iii) environmental related transactions (UNSD et al., 2014a).

With respect to the SEEA CF, we restrict our analysis to what the SEEA CF refers to as natural resources, and in particular to the compilation of asset accounts. “The scope of environmental assets measured in the SEEA CF is greater than the scope of environmental assets following the SNA definition of economic assets. This is because there is no requirement in physical terms that environmental assets provide economic benefits to an economic owner” (ref. paragraph 5.39 UNSD et al., 2014a). A few examples: remote land, barren land, inaccessible timber resources, known mineral deposits with no current economic value. The SEEA CF explicitly states that “quantities [of such assets] should be recorded separately from quantities of environmental assets that do deliver economic benefits to economic owners” (ref. paragraph 5.40 UNSD et al., 2014a).

**Fig. 2:**
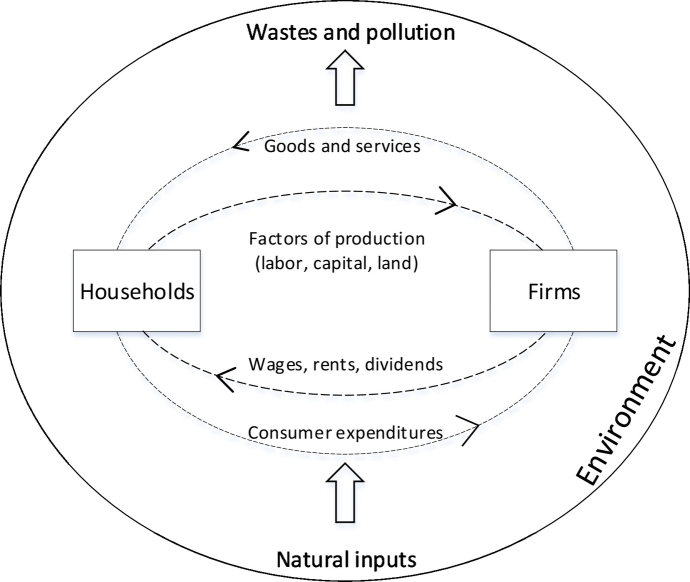
Simplified representation of the circular flow model and its relationship with the natural environment.

**Fig. 3:**
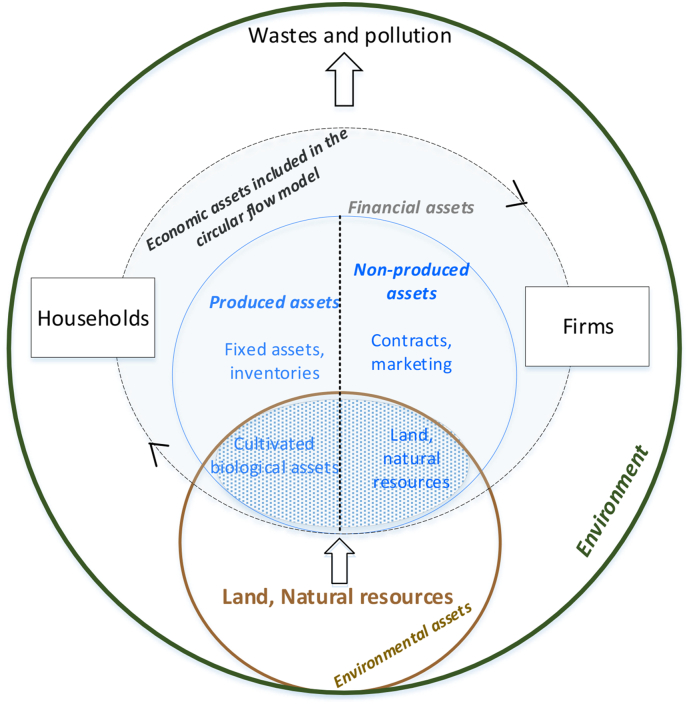
Linkages between environmental and economic assets.

In Fig. 3 the SNA simplified representation from Fig. 2 is expanded according to SEEA CF definitions.

In Fig. 3 economic assets explicitly refer to elements tabulated within the SNA, while environmental assets refer to elements tabulated within the SEEA CF. Economic assets in the SNA can be produced assets, non-produced assets, or financial assets. Fig. 3 shows that there is an overlap between economic and environmental assets, in fact some environmental assets already belong to the SNA as produced and non-produced assets. The difference between produced assets and non-produced assets lies mainly in the role of human inputs: cultivated biological resources are in fact both a produced economic asset in the SNA, and an environmental asset in the SEEA CF. This implies that natural inputs are provided *for free* to economic agents that use them to generate an economic product. For example, crops are part of the SNA as products generated by the agricultural sector for further transformation, domestic final consumption, or international trade; they are also part of the SEEA since they are biomass generated by natural processes. Non-produced assets are assets that were not generated through processes of economic production, and with a limited or null human input. For example, managed forests that generate an economic benefit because of timber harvest are accounted in the SNA (as economic assets) and in the SEEA CF (as environmental assets): the human input in this case lies in the management regime but not in the natural growing process. Remote forests do not generate any economic benefit and are not managed: they are only accounted in the SEEA CF as environmental assets, and not in the SNA. Table 1 summarizes these concepts.

Processed and adapted from Fig. 5.1 in UNSD et al. (2014a).

**Table 1:**
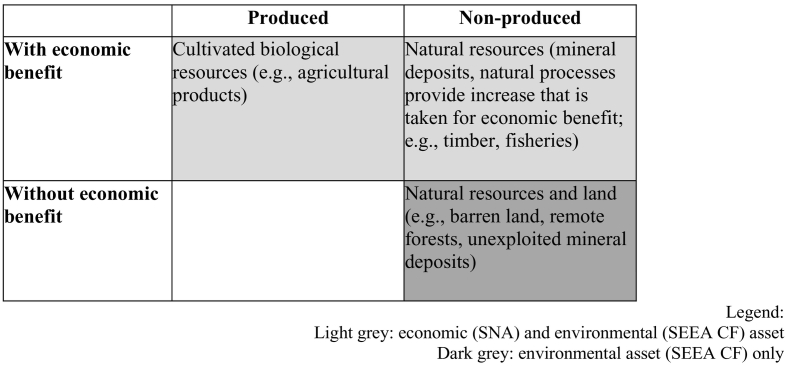
Classification of environmental assets in the SNA and in the SEEA CF.

Light grey: economic (SNA) and environmental (SEEA CF) asset.

Dark grey: environmental asset (SEEA CF) only.

The SEEA CF currently reports in Chapter 5 that it explicitly dedicates to asset accounts the following natural inputs: mineral and energy resources, land, timber, aquatic resources, biological resources, soil, and water. Environmental assets are addressed and accounted as individual components that make up the environment, mass and biomass, that can be harvested, extracted, or otherwise taken for direct use in economic production, consumption, or (owned) stock accumulation. Environmental assets are accounted as raw stocks of natural resources and not as media for ecological health and growth. In asset accounts an addition to a stock of resources (e.g., growth in stock and discoveries) is contrasted with a reduction in stock (e.g., extraction, natural and catastrophic losses) in order to calculate how much a total stock has changed at the end of an accounting period (usually one year). This kind of information is useful to check whether any resource is managed sustainably, i.e., whether periodic removal of biological resources exceeds natural rates of growth and replenishment.

### 2.3 SEEA EEA framework

The SEEA CF in principle makes it possible to track and manage natural resources as individual components. However excessive exploitation of resources can irreversibly damage an environmental asset, including the natural resource itself, and damage the larger ecosystem in which that resource resides or functions. For example, excessive timber removal can cripple a forest ecosystem's capacity to regenerate. The SEEA CF can report cubic meters of timber year after year, and this will likely show a dramatic decrease. But the SEEA CF cannot report the damage that propagates as the forest ecosystem tree cover is removed – damage to the hydro-geological equilibrium, to carbon sequestration and accumulation of biomass and soil, and to the visual appeal of the area for recreation activities. In the same way, pollutant accounts in the SEEA CF report data linked to specific economic activities, but these accounts do not provide information about how pollutant loads will affect the ecosystem, or whether the ecosystem is still capable of removing and absorbing them without irreversible degradation. The SEEA CF can provide some additional valuable information to the SNA, but not enough information to support a retroactive analysis of sustainable management of natural resources, or to infer correlations that could inform forward planning.

**Fig. 4:**
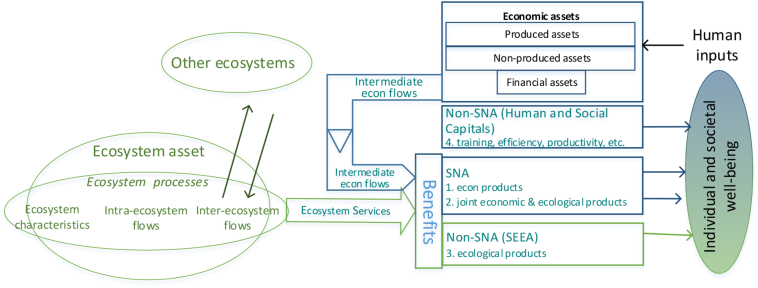
Stylized model of flows related to ecosystem services.

The SEEA Experimental Ecosystem Accounts (SEEA EEA, UNSD et al., 2014b) were developed to fill this important gap. Its theoretical framework will be more complex than the SEEA CF. The SEEA EEA has to deal with many factors not conventionally measured in economics, and beyond the accounting measures that the SEEA CF offers beyond the SNA. Ecology – a multifaceted family of natural sciences, spatial analysis, and conservation planning – will play a major role, since accounts will only be correct if what is biophysically assessed is properly measured. The SEEA EEA must include asset and flow accounts: ecosystems and the flows of ecosystem services that they generate. Fig. 4 shows an adapted version of the SEEA EEA theoretical framework (UNSD et al. 2014b) that depicts the relationship between ecosystem and economic assets, and further includes non-SNA benefits that contribute to human well-being without direct dependency on benefits from ecosystem services, for a more complete spectrum of SNA and non-SNA benefits in a single diagram.

Processed and adapted from UNSD et al. (2014a, 2014b).

According to SEEA EEA definitions, the equivalent of capital stocks in ecosystem accounting are ecosystem assets. Each ecosystem asset is a spatial area having a range of ecosystem characteristics, such as slope, altitude, rainfall, land cover, and biodiversity. In ecosystem accounting, ecosystem assets yield flows of two types. First, there are the intermediate flows within and between ecosystem assets that reflect ongoing ecosystem characteristics and processes (including both intra- and inter-ecosystem flows). Second, there are the ‘final’ flows from ecosystem assets to economic or individual use: ecosystem services. Flows of ecosystem services may relate either to natural inputs that flow from the environment to the economy, or to residuals generated by human activities that flow to the environment. Through the concept of ecosystem services, a link is established between ecosystem assets and the benefits derived and enjoyed by people. Thus in the SEEA EEA, ecosystem services are neither ecosystems nor final economic benefits, but flows connecting ecosystem assets to benefits.^9^ In the SEEA EEA design, ‘intermediate ecosystem services’ are flows between ecosystem assets and intra-ecosystem flows may not be classified or tabulated, because they are complex and very numerous, and many by definition are not ‘final’ flows to people.

The SEEA EEA includes extent and condition accounts that relate to the ecosystem as a whole, and Supply and Use tables that relate to ecosystem services. Future development of the system will include monetary asset accounts for ecosystems, and integration of ecosystem accounts and economic accounts in monetary terms.

## 3 The underpinning theoretical frameworks on the ecosystem services side

### 3.1 Cascade framework

So far we have only considered theoretical models on the accounting side. Other theoretical frameworks have been developed, and are referred to by researchers and practitioners working on natural capital and ecosystem services. The very popular frame reported in the Millennium Ecosystem Assessment (MA, 2005) has fed numerous initiatives, among which we mention The Economics of Ecosystems and Biodiversity (TEEB). In the ecological-economic foundation of TEEB (2010) it is possible to find one of the early versions of the cascade model, proposed by Haines-Young and Potschin (2010), and largely used in a variety of applications (Potschin and Haines-Young, 2016). The diagram separates: ecological structures from processes generated by living organisms, and ecosystem services from the benefits that people eventually enjoy. The presence of ecological structures has the functional capacity to provide services that humans find useful. The cascade model represents the theoretical basis of the Common International Classification for Ecosystem Services (CICES),^10^ often used as a reference by ecosystem services practitioners (Maes et al., 2014).

**Fig. 5:**
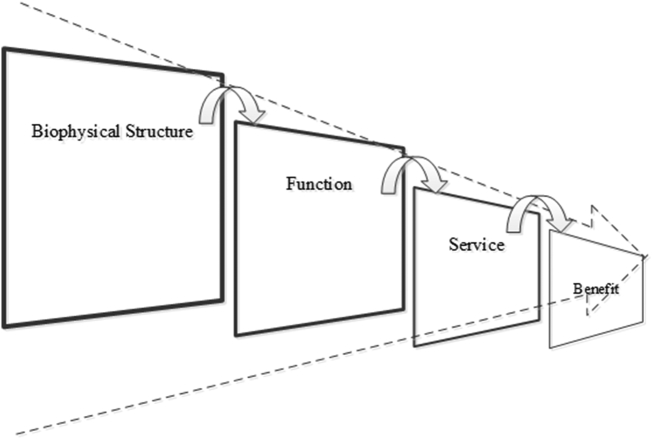
The ‘telescopic’ cascade model based on system ecology categories.

Source: La Notte et al. (2017b).

Using concepts from systems ecology (biomass, information, and interaction), it is possible to add a deeper ecological perspective to the cascade model (La Notte et al., 2017b). Because the elements of the cascade are not ‘equal’ in terms of ecological complexity, the causal sequence can be visually represented, with larger tiles representing higher systemic complexity (Fig. 5).

On the one hand, biophysical structure and function remain linked to the ecosystem perspective. Processes that take place at a deep systemic and holistic level, such as nitrogen and carbon cycling, primary production, and so on, are considered to be background and intermediate processes that occur on very large scales – creating or maintaining the ecosystem. On the other hand, ‘final’ ecosystem services can be identified as usually countable individual flows, where from an economic perspective each flow occurs on a smaller, human, scale. From systems ecology, interaction and information are complex processes that take place at higher hierarchical levels (i.e., refer to Function and Service in Fig. 5) and may not be directly perceived by humans, whereas mass and biomass are less complex elements that are more easily perceived by humans. Table 2 reflects an attempt to establish which step of the cascade the typologies of SEEA accounts (i.e., both the CF and the EEA) may refer to. Also Lai et al. (2018) investigated how the Cascade model can correspond to the SEEA accounts.

**Table 2:** Cascade model component terms and their proposed correspondence with the SEEA.

Term	Definition and examples	SEEA accounts
Biophysical structure	Biotic and abiotic components that provide the setting for ecosystem processes. Examples: terrain, weather, inland water bodies, forest tree cover	SEEA EEA extent and condition accounts
Ecological process /function	An ecological interaction involving biotic and abiotic components in an ecosystem over time. Processes may contribute to or create multiple ecosystem services. Examples: Net primary production, carbon cycling, nutrient cycling, hydrologic cycle	SEEA EEA condition accounts with links to capacity
Ecosystem service	A flow generated by ecological processes, that humans directly use or appreciate. Examples: wild pollination, water purification, aesthetic beauty of landscape, protection against the risk of flooding	SEEA EEA ecosystem services supply and use accounts
Benefit	Examples: natural resources for multiple uses, availability of water for multiple uses, enhanced personal well-being due to beauty of landscape	SNA and SEEA CF accounts eventually combined with SEEA EEA flows

As to ecosystem services (ES), it is important to highlight that supply and use tables report actual ES flows, i.e., only that ES potential which interacts with ES demand (La Notte et al., 2019). Supply tables show from which Ecosystem Types the ES actual flow comes from, and the use tables show to which economic sectors and households ES go. When ES actual flow is embedded in an SNA product, the contribution from Ecosystem types should be disentangled from the final product that also includes human inputs (as an example, ref. the chapters on crop and timber provision in Vallecillo et al., 2019).

CICES is an application of the MA 2005 four types of ecosystem services through the cascade framework, counting ‘supporting services’ as background/intermediate processes, and three major groups as ecosystem services: (i) provisioning, (ii) regulating and maintenance, and (iii) cultural. The details of the classification itself are available elsewhere, but importantly here, all the classified flows of ecosystem services can be final, depending on the context. It is in fact an accepted principle that intermediate ecological processes take place at the ‘function’ step (La Notte et al., 2017b), and that whether the ‘virtual’ transaction (in accounting terms) of any given ecosystem service is intermediate or final depends on the type of beneficiary and use, and not on whether it involves a good versus a process (Obst, 2016).

### 3.2 EPA frameworks

Important work on classification of the links between natural and human systems has been undertaken at the US Environmental Protection Agency (EPA), where two classification systems were developed.

**Fig. 6:**
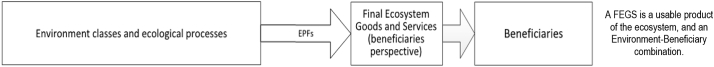
The FEGS-CS connection between the ecological and economic production functions.

Adapted from Landers and Nahlik (2013).

The concept of Final Ecosystem Goods and Services (FEGS) is defined as “components of nature, directly enjoyed, consumed, or used by human beings” (Boyd and Banzhaf, 2007), and is offered by scientists at the US EPA to define, classify, and help refine measurement of ecosystem services. Fig. 6 shows the theoretical framework underpinning the FEGS classification (FEGS-CS, Landers and Nahlik, 2013). Final ecosystem goods and services are identified by considering specific biophysical components of goods and services that are principally derived from nature within a landscape (environmental classes), and by determining the specific ecosystem goods or services that beneficiaries (beneficiary categories) value. The FEGS-CS classifies goods and services that are environmentally derived. This means that FEGS are produced through ecological production functions (EPFs), without major inputs of labor or capital from humans.

The FEGS-CS matches types of ecosystem goods and services from identified Environments to actual user/Beneficiary types.

**Fig. 7:**
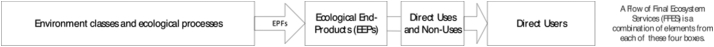
The four-part classification structure of the NESCS.

Adapted from USEPA (2015).

The National Ecosystem Services Classification System (NESCS) comprises four classifications – Environment, Ecological End-Products (EEPs), Uses, and Users. The NESCS begins with Environment classes from the FEGS-CS, but classifies the actual physical elements from natural products and processes that people use or appreciate (EEPs), and offers the flexibility of separately classifying Uses from Users. In fact, in the NESCS, different Users may employ the same Use, or any particular User may employ the same EEP to different Uses (Fig. 7).

The NESCS is built to be able to identify and classify any relevant flow of final ecosystem services that may enter any User's utility function (Industry, Household, or Government). A beneficiary perspective is introduced by the FEGS-CS but is fully developed within the NESCS. The ecological side in the two classification systems is about the same: ecological production functions describe processes by which one or multiple ecological end-products are generated, but there is no attempt to classify the myriad ecological processes necessary to generate any EEP. EPFs are embraced as offering the ability to characterize and gauge ecological ‘production’ dynamics for EEPs – so that people know what intermediate ecological processes to protect in order to have the ES they desire. Processes take place at the ecosystem level.

The CICES, the FEGS-CS, and the NESCS are currently being considered by experts working on the SEEA EEA as possible reference classifications for ecosystem services to be named in ecosystem services supply and use tables. A NESCS-like framework is being used provisionally for NCA work by a multi-agency, international, NGO, and private-sector team in the US exploring NCA accounting structures and attempting to match existing data to fill elements of those pilot accounts (Warnell et al., forthcoming).

## 4 Comparing classification and accounting theoretical frameworks

**Fig. 8:**
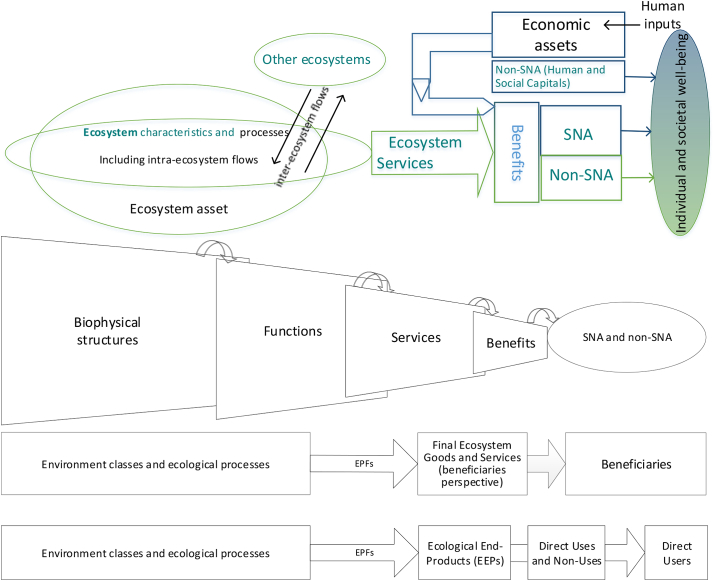
Comparison between the SEEA EEA theoretical framework and the three ecosystem services classification frameworks.

Having described the theoretical frameworks from both the accounting and the ecosystem-services sides, we now attempt to combine where the three models currently considered by the experts working on SEEA EEA are placed, compared with an accounting framework (Fig. 8).

Fig. 8 shows that the economic component can be explicitly included in the framework (FEGS-CS and NESCS) or be simply implied (cascade model).

In accounting terms, purely economic components and flows are recorded in the SNA. On the other hand, the SEEA EEA describes components and flows that involve ecological parts.

CICES explicitly considers the ecological production of Services, while FEGS-CS and NESCS classify the relationship between first what FEGS-CS calls ‘FEGS’ and NESCS calls EEPs, and second Use-User combinations (the economic element that FEGS-CS calls Beneficiaries). Specifically: Services are an identified step in the cascade model that CICES treats as the object of classification, whereas Final Ecosystem Services in the FEGS-CS and the NESCS connect the EEPs(/FEGS) to specific Environments and to specific Beneficiaries/Users, through use. FEGS-CS and NESCS sub-classify parts that together characterize flows of final ecosystem services: Environment and Beneficiary for FEGS-CS, and Environment—Ecological-End-Product—Use—User for NESCS. Treating Beneficiaries or Users as constituent components for defining ES more closely approximates pathways of SNA and non-SNA benefits that reach humans (who are producers, consumers and living organisms). More specific allocation of benefits for accounting purposes follow the beneficiary perspective of FEGS-CS and NESCS.

There is room to explore whether CICES, FEGS-CS, and NESCS might be considered as complementary classifications that focus on different steps of the general chain described by the cascade model. But the cascade model only implies specific human uses that national accounting seeks to account for. In accounting terms: the flow of the services is tackled in a less anchored way by CICES (only some Services seem to connect to uses directly), while the FEGS/Ecological End-Products and their use for individual and societal well-being are broken out specifically by FEGS-CS and NESCS. The latter classifications do indeed fill an information gap that arises between the SEEA CF and the SEEA EEA, concerning benefits associated with ecosystem services from named ecosystem assets, where the flows are economic inputs (SNA) or flow directly to people (non-SNA).

## 5 Connecting classification and accounting theoretical frameworks

In the SEEA EEA, ecosystem services are depicted through supply and use tables. Compared to the SNA, the production boundary has been broadened (Eigenraam and Obst, 2018; La Notte et al., 2019): additional rows reflect the flow of ecosystem services and additional columns name ecosystem units as the producers of flows (in the Supply table). Starting from the proposed Technical Recommendations (UN et al., 2017), we further expand the ‘product’ Use table by including not only SNA but also non-SNA benefits. At the moment, the SEEA EEA general frame considers SNA benefits to be ‘products’ with economic inputs and with or without ES inputs, and non-SNA benefits to be the corresponding ecosystem services that are consumed directly (like economic ‘products’, but without including substantial economic inputs) by an end-user (UN et al., 2014).

In order to supplement basic accounts with additional information, we could hypothesize that all benefits can be disentangled from services (both SNA and non-SNA benefits). By looking at services rows and at SNA and non-SNA benefits rows and their allocation to users, one may practically identify where the different classification systems find complementarity. For the sake of simplicity, we will now refer to CICES rev. 5.1 and to the NESCS four-part structure.

Among all the ecosystem services listed in CICES, we select a few and attempt a cross-classification with NESCS. Specifically: forage and wood biomass growing, wild pollination of crops, water purification, air filtration, carbon sequestration, flood and erosion control, and provision of outdoor recreation opportunities. For those ecosystem services, we proceed to identify the flow of the service, the benefit generated and the beneficiary.

To allocate a benefit to a beneficiary it helps to assign a specific use. By including Uses and Users in the classification of ecosystem services, NESCS offers a place to separate ecosystem service flows by Use to named economic units – enhancing the resolution for value assignment. This is exactly how we proceed in allocating the benefit: it would be possible to identify the use according to the user, or vice versa (Table 3).

**Table 3:** Complementarity between CICES and NESCS for selected ecosystem services.

CICES classification	*Re*-phrased as contribution of ecosystem	NESCS		
		Ecological End-Products [SNA and non-SNA Benefit]	‘Use’	NESCS 'User’Beneficiary
Animal husbandry	Forage biomass growing	Flora ([2(2/4/5)].2.)^a^ [SNA: livestock]	Support of animal cultivation (.1105.)	Agriculture (111) [2(2/4/5)].2.1105.111
Materials from plants	Wood biomass growing	Flora ([2(1/2)].2.) [SNA: timber]	Raw material for transformation (.1101.)	Agriculture (111) [2W].2.1101.111
Pollination and seed dispersal	Wild pollination of crops	Fauna ([2(2/3)].3.) [non-SNA: wild pollinators of crops]	Support of plant or animal cultivation [for Agriculture, households] (.1105.)	Agriculture (111) [2(2)].3.1105.111 Households (2) [2(3)].3.1105.2
Dilution by freshwater ecosystems	Water purification (nitrogen removal)	Water ([1(1/2/3/6)].1.) Liquid water ([1(1/2/3/6)].12.) [non-SNA: cleaned water]	Support of plant and animal cultivation (.1105.) Distribution to other uses (.1104.) Support of human subsistence (.1106.)	Agriculture (111) [1(1/2/3/6)].12.1105.111 Waste mgmnt (156) [1(1/2/3/6)].12.1104.22131 Households (2) [1(1/2/3/6)].12.1106.2
Global climate regulation	Carbon sequestration	Combined End-Products (.8X.), Regulation of Extreme Events (.82.) ([WW].82.) [non-SNA: complex biological growth that newly sequesters carbon]	Protection or support of human health and life (.1205.)	All: [WW].82.1205.1(/2/3)
Filtration by plants	Air filtration	Combined End-Products [2(1/2/3/4/5)].82) [non-SNA: complex of biological elements that clean air]	Protection or support of human health and life (.1205.)	Households (2) [2W].82.1205.2
Control of erosion rate	Erosion control/soil retention	Soil ([2(1/2/3/4/5)].6.) [non-SNA: soil tilth that holds against erosion]	Support of plant cultivation (.1203.)	Agriculture (111) [2W].6.1203.111
Flood protection	Flood control	Regulation of extreme events ([2(1/2/3/4)].82.) [non-SNA: complex of biological elements that retain or slow water] [SNA]	Protection of human life (.1205.) Protection of human property (.1206.)	Households (2) [2W].82.1205.2 Industry (1) [2W].82.1206.1 Households (2) [2W].82.1206.2
Interactions with natural environment	Provision of outdoor recreation opportunities	-Scapes ([WW].81.) [non-SNA] [SNA]	Recreation (.1207.) Tourism (.1207.)	Households (2) [WW].81.1207.2 Accommodation and Food services (172) [WW].81.1207.172

a NESCS uses codes that correspond with choices from each of the four classifications as each is fully listed in the NESCS Four-Part(/Group) Structure (not presented in this paper). So WW.XX.YYYY.ZZZ represents places for 1 to 7 digits by Environment(WW)–Ecological End-Product(XX)–Use(YYYY)–User(ZZZ). All four places must be represented for a full code representing a potential FFES. In Table 3, external parentheses and brackets are used to isolate partial codes, alternative choices are represented using internal parentheses and slashes, and letters from the representational code (W.X.Y.Z) are used to show that there is a range of alternate choices where that letter is. Thus ‘([2(2/4/5)]’. means that the Environment is terrestrial (2), and an Agroecosystem (22), Grasslands (24), or scrubland (25). The EEP code (.2. for flora), Use code (.1105. for support of animal cultivation), and the User code (.111 for commercial agriculturalist) are then concatenated to build the first row. ‘2W’ means any terrestrial environment, and later in the table ‘WW’ then means any environment.

By inserting the Use and User categories it is possible to more clearly assign the benefits from ecosystem services. A desirable feature of the use table can be inclusion of Uses as sub-categories within the standard classification of economic units (Users), in order to provide clearer and transparent paths of allocation.

A few comments on the examples reported in Table 3. The difference between SNA and non-SNA can be derived from and depicted using the Use description: whenever the use is ‘Raw material for transformation’ or ‘Direct consumption’ by an Industry and not a Household, it will always be an SNA benefit. In some cases we may find a difference in the definition of benefit, as in the case of ‘biomass growing’ and ‘soil retention’: the former is an SNA benefit because it enters into an SNA product (forage for livestock in the case of animal husbandry, wood in the case of material from plants); soil retention can have on-site effects which could impact soil fertility (and thus agricultural production), and off-site effects (i.e., sedimentation) relevant to a non-SNA benefit. In other cases the definition of the EEP/benefit category is the same (e.g., water), but the difference can be read in Use and Beneficiaries: cubic meters of water as a benefit from water provision (or water regulation) would include all water users (energy [Use=1102], industrial processing [Use=1103], etc.); less nitrogen per cubic meter of water only includes those uses that require clean water (reported in Table 3). The former is quantity of water (SNA benefit), while the latter is quantity of ecologically-purified water to a certain quality (non-SNA benefit).

There are other cases where the benefit can be both SNA and non-SNA depending on the use. It happens for ‘Regulation of extreme events’ where both the ‘Use’ and ‘Beneficiary’ categories differ: whenever the use concerns privately-owned properties, it is about SNA benefits; whenever the use concerns human life, it is about non-SNA benefits. Similarly for ‘Provision of outdoor recreation opportunities’, when the emphasis is more on recreation that is not mediated by a business or institution, it is about non-SNA benefits, and the beneficiaries are households. This is the same use by different beneficiaries. When the emphasis is more on tourism and related businesses (‘Accommodation and Food Services’), Education (‘Education’), and the ‘Arts’ (‘Arts and Recreation’), the recreation beneficiaries are associated more often with conventional SNA products(/benefits).

Each time ES accounting applications take place, a choice is made for how to interpret ecosystem services, ecological processes, and benefits, and for how to frame their users in the use table. For example, in KIP-INCA, when accounting for outdoor recreation (Vallecillo et al., 2018) the opportunity to enjoy nature-based recreation (interaction with natural environment) on a daily basis is separated from nature-based tourism, i.e., ‘recreation’ is attributed to households as final beneficiaries. Another example: flood control (Vallecillo et al., 2019) as protection of human property is attributed to both economic sectors (i.e., agriculture, manufacturing and energy production, construction, transport, waste management and other tertiary) and households (residential use).

The research on refining the comparison and connection between classifications and accounting frameworks inevitably develops throughout application.

## 6 Conclusion

The concept of ecosystem services is a viable tool for impact assessments, because it reports “unattended and unintended consequence of policy implementation on human well-being” (Kumar et al., 2013). The goal to integrate ecosystem services into conventional policies can be addressed via integrated accounting systems.

The need for an economic accounting system to be integrated with not only ‘environmental’ satellite accounts but also with ‘ecosystem’ and ‘ecosystem services’ satellite accounts is acknowledged by the numerous initiatives undertaken by international and national organizations, institutes, and agencies. Traditional accounting of economic systems fails to account for the hidden dependency of economic sectors and households on nature, by only considering individual natural resources and not the dynamic, interactive, and generating processes that take place in ecosystems. No sustainable assessment and planning could be undertaken by policymakers without this broader perspective.

Although a considerable amount of work is in progress at different scientific and organizational levels, it is hard to find an agreed common ground where all definitions and classification concepts are harmonized. Since concepts should ideally be set before any definition and classification can be finalized, we attempt to investigate: (i) the conceptual notions behind the accounting side, and (ii) the conceptual notions behind the ecosystem-services side, in order to find out (iii) how the two conceptual systems compare and can eventually fit.

What emerges from considering the accounting frameworks with the goal of integrating accounts is that:

• environmental assets in the SEEA CF include produced (cultivated biological assets) and non-produced (natural resources and land) assets that are used by economic sectors within the SNA;
• ecosystems and ecosystem services are tackled as new and separate accounts by the SEEA EEA;
• ecosystem services are accounted through the supply and use tables, which can report services and products;
• there is currently a lack of continuity between the SEEA CF and the SEEA EEA in terms of services, benefits, and products;
• disentangling ecosystem services (as ecosystem contributions) from benefits will assist in the task of enlarging the ‘product’ section in the supply and use tables, in order to account not only for SNA benefits but also for non-SNA benefits.

What emerges from considering the ecosystem services frameworks with the goal of integrating accounts is that:

• the focus is supposed to be on distinguishing, naming, and hierarchically classifying flows from ecosystems to humans;
  • separating intra- and inter-ecosystem flows (ecological characteristics and processes) from those that humans directly use or appreciate matters for measurement, and the complexity of ecology and of potential flow paths demands classifications that help ecosystem services and natural capital accounting practitioners separate them as clearly and as consistently as possible.

Based on these statements:

1. the ‘telescopic’ cascade framework is able to reflect the conceptual content and complexity of ecosystem services as ecological flows but is not meant to explore the ‘beneficiary perspective’;
2. there is a need to complement the SEEA EEA with the SEEA CF in order to clearly separate ecosystem services (where transactions take place) from natural assets (i.e., mass and biomass), and to properly expand supply and use tables to include SNA and non-SNA benefits.

This requires disentangling ecosystem services from benefits – the two notions can neither be confused nor used interchangeably in a theoretically correct practice.

Moreover, it is also possible to state that:

3. in order to properly identify the precise ecosystem services that beneficiaries(/users) value, it is necessary to discriminate among different uses;
4. the FEGS and NESCS bring structure and rigor to uses and beneficiaries
5. CICES, FEGS, and NESCS, despite obvious structural differences, are largely not in conflict but rather complementary, because each element in the chain comprising the path from ecosystems to final beneficiaries does indeed need its own classification.

These statements may be validated, updated, or restructured following case applications and can contribute to the debate over the SEEA EEA technical revision that is currently taking place.

## Footnotes

1 https://www.wavespartnership.org/en

2 http://ec.europa.eu/environment/nature/capital_accounting/index_en.htm

3 http://biodiversity.europa.eu/maes

4 https://ensym.dse.vic.gov.au/home/aboutensym

5 http://www.statcan.gc.ca/pub/16-201-x/2013000/aftertoc-aprestdm1-eng.htm

6 http://www.minambiente.it/comunicati/il-secondo-rapporto-sullo-stato-del-capitale-naturale-italia

7 https://www.gov.uk/government/groups/natural-capital-committee#role-of-the-group

8 Final ecosystem services are directly used or appreciated (as SNA or non-SNA flows).

9 For SEEA EEA accounting, a ‘benefit’ from an ES is a) an input to economic production, or b) an input to an individual's basket of goods and services for direct consumption. This breaks from economists' convention of defining a ‘benefit’ as a change in the individual's welfare, often represented as a ‘willingness to pay’ for a good or service, or a change in their ‘basket’. The difference follows in part from accountants not dealing with welfare or welfare questions as they attempt to accurately characterize stocks and flows in an accounting period, and in part from accountants dealing with transaction costs, and not consumer surpluses that are part of welfare calculations.

10 http://cices.eu/
